# Predictors of Social Exclusion among Adolescents: The Weight of Physical Self-Concept Dimensions

**DOI:** 10.3390/children11101235

**Published:** 2024-10-14

**Authors:** Rosa González-Delgado, Antonio J. Rodríguez-Hidalgo, Rosario Ortega-Ruiz, Juan de Dios Benítez-Sillero, Javier Murillo-Moraño

**Affiliations:** 1 Department of Education, Regional Government of Andalusia, 41071 Seville, Spain; m42goder@uco.es; 2 Department of Psychology, University of Cordoba, 14071 Cordoba, Spain; ed1orrur@uco.es; 3 Department of Specific Didactics, University of Cordoba, 14071 Cordoba, Spain; eo1besij@uco.es; 4 Research Group in Sport and Physical Education for Personal and Social Development, 14071 Cordoba, Spain; z02mumoj@uco.es; 5 Teacher Training College “Sagrado Corazón”, University of Cordoba, 14006 Cordoba, Spain

**Keywords:** subtle exclusion, manifest exclusion, victimization, adolescents

## Abstract

**Background:** Social exclusion victimization among adolescents causes significant damage and harm to development and social balance. Many of the aggressions that lead to this are based on social stigmas, particularly related to physical appearance in school settings involving physical and sports activities. This study investigates the relationship between victimization through social exclusion (both manifest and subtle forms) and physical self-concept, specifically examining ability, attractiveness, physical condition, and strength. **Methods:** A total of 876 adolescents (mean age = 14.91; standard deviation = 1.71 years), evenly divided between boys and girls, from secondary schools in Andalusia, Spain participated. They completed a self-report questionnaire assessing physical abilities, attractiveness, physical condition, and self-perceived strength, alongside an instrument measuring experiences of social exclusion and manifest exclusion. **Results:** Our findings indicate that physical ability, attractiveness, and condition are negative statistical predictors of both manifest and subtle exclusion victimization, while self-perceived strength is a statistical predictor of subtle exclusion. Manifest exclusion impacts both genders similarly, but girls are more vulnerable to subtle forms of exclusion. Regardless of gender, physical ability and attractiveness significantly predict both types of exclusion. **Conclusions:** Our results highlight the importance of physical self-concept for wellbeing and maintaining self-concept balance. The inclusion of interventions addressing social exclusion in physical education is crucial, particularly those that work to mitigate social stigmas against adolescents who struggle in physical or sporting activities. A gender-sensitive approach should also be incorporated. The growing field of research on adolescent social exclusion, both manifest and subtle, underscores the need for further exploration of its links to physical condition, physical activity, self-perception, and societal stereotypes.

## 1. Introduction

Adolescence is a critical phase in the life cycle during which young individuals actively seek to engage in activities and spend time with their peers to feel integrated within a group. Physical activity, whether in school or extracurricular settings, serves as a prime environment for adolescents to not only satisfy their strong social drive [1] but also to explore their self-identity, understand others, and develop essential social skills [2]. The quality and availability of these experiences play a critical role in shaping both individual and social development during adolescence. On a personal level, they contribute to the progression of self-concept and the establishment of positive self-esteem. Socially, these experiences foster improved communication skills, the formation of emotional bonds such as friendships, and the ability to adopt various social roles while adapting behavior to meet group expectations. This comprehensive process facilitates the development of social competence, as well as the stabilization and adjustment of personality during a particularly sensitive and demanding stage of life [1,3].

However, the processes of social interaction among adolescents do not always generate healthy experiences and bonds, leading to problems and harm that condition their identity development and emotional well-being [4]. Victimization among peers is recognized as a public health problem [5,6]. In this regard, the general phenomenon of bullying stands out as the focus of a set of aggression and victimization problems that include various forms of stigmatization of a boy or girl by their peers. It refers to the harm a student suffers from their peers, intentionally, repeatedly, under a power imbalance, and through the perversion of the elementary principles of moral reciprocity [7]. This harm can not only be inflicted through clearly observable actions but can also occur through the omission of actions and active exclusion. It can also be subtly slid into social relationships that take place in the context of common activities. It is a type of discriminatory and/or exclusionary victimization [8,9] that manifests very clearly as a shared belief in the devaluation and rejection of a peer or as a subtle devaluation of that peer’s competence, which can lead, consequently, to an avoidant or isolating attitude towards them. Among adolescents, victimization occurs largely through a relational aggression that generates social exclusion [10]. Relational aggression is often: (a) direct, where aggressors exclude the victim through explicit actions; or (b) indirect, where aggressors socially ostracize or deliberately ignore the victim, or isolate them from their peers through spreading rumors behind their back via the peer network to damage their image. Thus, social exclusion—whether more evident or more concealed—constitutes both a means and an end for these actions or an omission of actions among peers. It is a form of victimization that generates emotional imbalance, loss of well-being, low self-esteem, loneliness, stress, anxiety, and depression in the adolescents who experience from it. This seems to lead to demotivation and low academic performance, absenteeism and/or the dropping out of school, an increased risk of addictions, an increase in aggressive behavior, suicidal ideation, and even suicide [11,12]. Furthermore, there is considerable research on the long-term consequences of social exclusion during adolescence, especially its impact on adulthood. Studies show that adolescents who experience social exclusion are at a heightened risk of developing mental health issues, which can persist into adulthood [13,14]. These individuals often struggle with social integration, lower life satisfaction, and reduced well-being as adults. Additionally, social exclusion can affect decision-making processes, increase risk-taking behaviors, and lead to substance abuse in adulthood. Research also highlights how persistent social exclusion in adolescence can foster negative self-concepts and even lead to physical symptoms like chronic pain or stress-related conditions.

On the other hand, although physical and sports activities can be a vector for educational and social inclusion, the very nature of these activities requires boys and girls to have attitudes of personal assertion, high motivation to master or overcome obstacles, and a certain degree of self-confidence. When some of the social benefits that the group provides to the individual are altered or severely impaired, as occurs in social exclusion, the necessary self-esteem, motivation, enthusiasm, and positive expectations required by physical activity and sports can become vulnerable in children and adolescents who suffer from these experiences of rejection or isolation victimization [15,16,17]. Some research concludes that there is an association between lower physical conditions and being rejected—manifested in social exclusion—in the practice of physical or sports activities, especially in competitive and team-based ones [18,19]. For example, in the study by Faith et al. [20], it was observed that three out of four students were excluded from games and sports due to their body size. On the other hand, other studies provide evidence of hidden social exclusion among peers in sports practice, due to the perception that the victim is outside the normative group because of differences in the level of specific physical skills they exhibit; or their executive competence; or their access to, and mastery of, materials or equipment, among other factors [21].

Undoubtedly, when this exclusion becomes shared within reference groups, it reinforces a form of social stigma closely tied to the standards of appearance, aesthetics, and physical or bodily competence, which are highly influential during adolescence [21,22,23,24]. For instance, lower proficiency in motor skills has been associated with difficulties in peer relationships and an increased likelihood of experiencing social isolation, with this relationship being more pronounced among boys than girls [25,26]. On the other hand, girls appear to be more vulnerable regarding their self-perception of physical attractiveness, often showing a greater tendency than their male counterparts to perceive themselves as overweight [22].

### 1.1. Social Exclusion among Schoolchildren

Social exclusion among peers is a form of psychological and social violence caused by actions and/or omissions of actions that transgress the relational moral principles [27,28,29] necessary for groups to learn how to interact together. Discrimination, when it involves mistreatment or violence, generates and is based on social stigmas that lead to social marginalization [30]. The aggressor or aggressors of this type of violence stigmatize and exploit the differences of an individual in relation to the rest of the group, whether based on ethnic–cultural differences, sexual orientation, gender identity, disability, physical appearance, or aesthetics, among others. Boys and girls in the educational system who are considered to have special educational needs (SEN) are at greater risk of being discriminated against and socially excluded [9,31]. In this regard, sports and physical activity are fertile grounds for this type of social discrimination [32,33].

Although social exclusion is a multifaceted phenomenon [34], from the perspective of the schoolchild who suffers this discrimination [11] scientific literature seems to converge on the existence of two major subtypes: (a) victimization by active, direct, explicit social exclusion through rejection, where the victim is clearly and directly shown that they are not welcome or wanted in the group; and (b) implicit, through ostracism and isolation and other subtle forms of aggression where the victim feels socially ignored [35,36]. Both subtypes of social exclusion are more frequently suffered by people who are also part of minority groups, which tend to be stigmatized as such groups [37] due to their ethnic-cultural origin, disability, or sexual orientation, among others. Both manifest and subtle forms of social exclusion have been documented among schoolchildren based on their physical appearance during physical activities. For example, it has been observed that three out of four students have witnessed how their overweight or obese peers have been ignored, avoided, and subjected to negative rumors—subtle social exclusion—or rejected and excluded from social activities—manifest social exclusion [38]. However, subtle social exclusion is more difficult to recognize, as it is largely suffered through microaggressions. Examples of subtle social exclusion include avoidance, deliberately not being physically close to the victim, avoiding verbal and eye contact, refusing to share academic or sports activities, mocking their appearance, or spreading rumors and defamatory comments behind their back, among others [27,31].

A review of the scientific literature on social exclusion reveals that, despite profiling two subtypes—manifest and subtle, as previously described—there is a lack of instruments that record both subtypes. Currently, the study by González-Delgado et al., 2024 (in press) [39] aims to validate a self-report scale that is sensitive to manifest and subtle social exclusion victimization in pre-adolescents and adolescents.

### 1.2. Physical Self-Concept

Body image is the self-conscious representation of one’s own body, the value attributed to it, and the confidence that one’s body will be functional, operational, and sufficiently well accepted by others [40]. In this sense, physical self-concept is closely related to general self-concept, identity, and self-esteem [41,42]. However, self-image, like any image, is a product of thought that fluctuates in its assessment. Just as self-esteem is nourished by the esteem, consideration, and respect that others have for us, the contempt and devaluation that peers direct at our person, and therefore our body, directly impacts self-assessment. As Fox [43] states, Physical Self-Concept is a “public” Self; a means by which we present ourselves to others and which also includes how the individual feels they are perceived and evaluated. There is evidence that schoolchildren considered unattractive or that have minor physical defects are more rejected and even excluded by their peers when it is perceived that they make no effort to improve their appearance [44]. A particular but widespread case in the Western world is the effect of being overweight. The confluence of being overweight and negatively self-perceiving one’s physical appearance—body dissatisfaction—in adolescents tends to impact the physical, social, and psychological aspects of an adolescent’s personality [45]. Some studies indicate that overweight and/or obese students may be more involved in bullying, either as victims, being more subject to mockery and verbal offenses than the rest of their peers [22,46], or in a mixed aggressor–victim role. At the same time, a good self-image—a positive self-perception of oneself—seems to mitigate the effects of being overweight and enhance satisfaction with one’s own body [22,46,47], acting as a protective factor against bullying.

Physical self-concept is defined as the perception that a person has of their appearance and physical abilities and is considered a very important domain in general self-concept [48]. A significant association between physical activity and physical self-concept and its various domains has been consistently demonstrated in both children and adolescents [49]. Analysis of self-concept dimensions [50] has revealed a multidimensional and hierarchical model that, fueled by self-esteem, has been established as a construct of four dimensions: physical abilities, physical condition, physical attractiveness, and physical strength. Abilities are considered physical skills. Physical condition is conceived as bodily functions and state. Physical attractiveness refers to the aesthetic appearance of the body. Physical strength is considered the capacity and control exercised over physical objects.

Studies reveal that adolescent girls generally have a poorer physical self-concept than their male peers [49,51]. Boys excel in strength, speed, endurance, and coordination, while girls excel in flexibility [52,53]. It has also been observed that girls are more likely to describe their weight in terms of being overweight, which is also related to the self-perception of physical attractiveness [22]. Research has shown that gender differences in self-perception are more significant than differences in objective measurements of performance and physical competence. This seems to suggest that gender differences in physical self-concept are not due to differences in motor or sports tests, but rather to gender stereotypes.

Body satisfaction positively influences physical self-concept [54]. However, throughout adolescence, levels of body dissatisfaction tend to be higher than at other developmental stages; with girls being more prone to record lower levels than boys [55]. This same gender trend is observed in relation to self-esteem [56,57,58].

### 1.3. Victimization by Social Exclusion among Adolescents and Physical Self-Concept

A review of the scientific literature shows that there is a vast area of knowledge yet to be explored regarding the possible relationships between peer social exclusion victimization and physical self-concept. Some studies are beginning to provide indications of the specific weight that physical self-concept holds in schoolchildren in relation to social exclusion [57]. However, the results from these studies are not conclusive for various reasons; it is important to note that these studies have measured social exclusion with a single item, as a specific form of bullying victimization. Another reason for scientific concern is that the samples used in these studies tend to be small and are not always representative of the reference adolescent population in each context. Nevertheless, some of the most relevant contributions of these investigations are highlighted.

The study of the relationships between bullying victimization and the victims’ physical appearance or aspect concluded that direct social exclusion is related to negative levels regarding their physical self-concept [59,60,61] For example, it has been observed that bullying victimization had an inverse relationship with self-perception of physical appearance, athletic competence, or body dissatisfaction. The study by Benítez-Sillero et al. [62] indicates that a positive physical self-concept can reduce the inhibiting effect on participation in physical activities of adolescents who are victims of bullying or cyberbullying. The systematic review by Arcila-Arango et al. [63] concludes that improving self-concept through physical education classes can decrease bullying victimization.

### 1.4. The Present Study

Given that peer social exclusion victimization is a complex and multidimensional construct, as it is generated through various stigmas and involves multiple aspects of the social dynamics and personality of the schoolchildren involved, as well as being highly impacted by the settings, contexts, and social origins of those affected, a previous study [39], developed a sensitive, reliable, and comprehensive instrument for measuring this phenomenon, which has allowed for a clearer establishment of the construct. Based on the same sample, the present study has the following objectives:

1. To identify the possible relationships between the levels of manifest and subtle social exclusion victimization, and the levels of the four domains of physical self-concept (ability, condition, attractiveness, and strength).
2. To determine whether the levels of the four domains of physical self-concept and age are statistical predictors of manifest and subtle social exclusion victimization.
3. To examine whether there are gender differences in the levels of manifest and subtle social exclusion victimization and in the statistical predictors of both subtypes of exclusion.

This research aims to test the following hypotheses:

**Hypothesis 1.**  *The four domains of physical self-concept are negatively related to the level of manifest social exclusion victimization.*

**Hypothesis 2.**  *The self-perceived levels of ability and physical condition are negatively related to the level of subtle social exclusion victimization.*

**Hypothesis 3.**  *The self-perceived levels of ability, condition, attractiveness, and physical strength are negative statistical predictors of manifest and subtle social exclusion victimization.*

**Hypothesis 4.**  *Especially in girls, the self-perceived level of physical attractiveness is a negative statistical predictor of manifest and subtle exclusion victimization.*

## 2. Materials and Methods

### 2.1. Participants

A total of 876 adolescents aged 12 to 19 years (mean = 14.91; standard deviation = 1.71 years), from four high schools in Andalusia, Spain participated in the study. A total of 48.7% of them were girls and 51.3% were boys, The sample was selected by convenience.

### 2.2. Procedure

A cross-sectional descriptive study was performed. The study was performed after obtaining the respective permissions from the participants’ educational centers as well as informed consent forms signed by their families. All national and international ethical standards were followed, such as the Declaration of Helsinki and personal data protection laws. Likewise, the project was approved by the Human Research Ethics Committee of the University of Cordoba. The participating students were informed of the objective of the study and the anonymous, confidential, and voluntary nature of their participation in it was highlighted.

### 2.3. Instruments

#### 2.3.1. Social Exclusion

An ad-hoc questionnaire was designed [39], which included a series of questions related to sociodemographic aspects (e.g., educational center, academic year, and sex), as well as questions investigating possible experiences of social exclusion experienced in the social environment of their reference groups (school, classroom, classmates, and friends) (Appendix A). The variables related to the experiences of social exclusion were measured using a Likert-type scale with 10 items and four response options, ranging from 1 (strongly disagree) to 4 (strongly agree). All items are written from the victim’s point of view, starting with headings such as: “I feel that …” “My classmates …” The internal consistency index shows satisfactory levels of reliability for the instrument (α = 0.932) and for each of its dimensions: (a) Manifest exclusion (ExN) = 0.906; and (b) Subtle exclusion (ExS) = 0.896.

#### 2.3.2. Physical Self-Concept

The abbreviated eight-item format [64] of the Physical Self Concept Questionnaire [65] was used in the study. Participants responded to the questionnaire using a five-point Likert-type scale, where 1 represented “false” and 5 represented “true”. Several sentences were used, for example, “I feel happy with my body image”. The scores from the individual items were summed to obtain an overall score on general self-concept, although items five and seven were swapped. The questionnaire assessed physical self-concept across four dimensions: strength, attractiveness, ability, and condition. The internal consistency values of the test were found as α = 0.81.

### 2.4. Statistical Analysis

Bivariate correlation analysis was conducted using Spearman’s test. Student’s *t*-test was used for comparisons by sex. To determine the relationships between the physical self-concept, age dimensions, and the exclusion variables multiple linear regressions were carried out. Data coding and analysis were carried out using the Statistical Package for Social Sciences (SPSS), version 28.

## 3. Results

Table 1 presents the linear regression models where manifest exclusion and subtle exclusion are the dependent variables, while age, PC Skill, PC Physical Condition, PC Attractiveness, and Strength act as independent variables. The model for manifest exclusion explained 5.1% of the variability in manifest exclusion, and the model for subtle exclusion explained 10.3%.

In both models, the predictors PC Attractiveness, PC Skill, and PC Physical Condition showed negative relationships with the dependent variables, suggesting that higher levels of attractiveness, skill, and physical condition in physical activity are associated with lower levels of both manifest and subtle exclusion. Additionally, in the subtle exclusion model, the predictor PC Strength showed a positive relationship, indicating that higher levels of strength are associated with higher levels of subtle exclusion.

Table 2 presents the Spearman correlations between age, manifest exclusion, and subtle exclusion with the various variables of Physical Self-Concept (PC): skill, physical condition, attractiveness, and strength. A negative association was observed between age and subtle exclusion, as well as with PC Attractiveness and PC Physical Condition. Manifest exclusion showed a positive correlation with subtle exclusion, indicating that those who experience manifest exclusion also tend to perceive greater subtle exclusion. Additionally, negative relationships were found between manifest exclusion and PC Attractiveness, PC Skill, and PC Physical Condition, suggesting that manifest exclusion is associated with lower perceptions of attractiveness, skill, and physical condition in physical activity. Lastly, subtle exclusion also showed negative associations with PC Strength, PC Attractiveness, PC Skill, and PC Physical Condition.

Table 3 and Table 4 present the linear regression models for the dependent variables subtle exclusion and manifest exclusion, respectively, differentiated by gender. The independent variables are the same as those used in Table 1: age, PC Strength, PC Attractiveness, PC Skill, and PC Physical Condition.

In the manifest exclusion model shown in Table 3, it was found to explain 7% of the variance in boys and 3.2% in girls. For this model, having lower PC Skill, in both boys and girls, is related to higher manifest exclusion. Additionally, PC Attractiveness also negatively predicted manifest exclusion in boys, but was not significant in girls.

Table 4 shows the subtle exclusion model, explaining 9.9% of the variance in boys and 9.5% in girls. In this model, both PC Attractiveness and PC Skill were significant negative predictors for both genders. Additionally, in boys, PC Physical Condition also acted as a negative predictor, meaning that poorer physical condition would imply greater subtle exclusion. However, the importance of the PC Attractiveness predictor was lower in boys compared to girls in this model.

Table 5 analyzes the differences between manifest exclusion and subtle exclusion based on gender. It shows that girls experience greater subtle exclusion compared to boys. However, no significant gender differences were found for manifest exclusion.

Table 6 and Table 7 present the stepwise linear regression models for the dependent variables manifest exclusion and subtle exclusion, respectively, differentiated by gender. The independent variables used are the same as those used in Table 6: age, PC Strength, PC Attractiveness, PC Skill, and PC Physical Condition.

Table 6 shows the manifest exclusion model differentiated by gender. For boys, in the first step, the negative predictor PC Skill explains 6% of the variance in the model. In the second step, the negative predictor PC Attractiveness is included, increasing the variance explained by the model to 7%. This suggests that, in addition to skill, attractiveness also contributes to manifest exclusion, providing additional explanation for the variance in the levels of exclusion observed in boys.

For girls, only one step is shown in the regression analysis, where the negative predictor PC Skill explains 3.2% of the total variance in the model. This indicates that, for girls, skill has a lesser ability to explain manifest exclusion compared to boys.

The subtle exclusion model, differentiated by gender, is presented in Table 7. For boys, the model showed that in the first step, the inclusion of the negative predictor PC Skill explained 7.3% of the variance. This suggests that lower skill was associated with higher levels of subtle exclusion. Adding the negative predictor PC Physical Condition in the second step increased the explained variance to 9.3%. Finally, the inclusion of the negative predictor PC Attractiveness in the third step raised the model’s total explained variance to 10%.

In contrast, the analysis for girls showed a different pattern. In the first step, the negative predictor PC Skill explained 6% of the model’s variance, indicating that although skill was also relevant for girls, its impact on subtle exclusion was less compared to boys. When the negative predictor PC Attractiveness was added in the second step, the explained variance increased to 8.5%, suggesting that attractiveness played a more significant role in girls than it did for boys. In the final step, the inclusion of the positive predictor age increased the total explained variance of the model to 9.1%.

## 4. Discussion and Conclusions

A review of scientific literature has revealed that some studies indicate a negative relationship between peer rejection and both physical fitness and body size [18,19,20]. Consequently, the first hypothesis in this study predicted that victimization due to manifest exclusion would negatively correlate with the four domains of physical self-concept. However, the results show that such victimization is negatively related to ability, physical condition, and attractiveness but does not affect strength, confirming the hypothesis in part. It was also found that manifest exclusion correlates positively with feelings of subtle exclusion. These findings provide further insights beyond previous research. The aspects of physical self-concept most closely associated with experiences of manifest rejection in adolescents are ability, physical condition (fitness), and attractiveness (physical appearance). Notably, strength is the only dimension of physical self-concept that does not appear to be related to victimization through manifest social exclusion. Furthermore, it has been observed that victimization due to subtle exclusion was negatively related to all four domains of physical self-concept—ability, condition, attractiveness, and strength—and was positively correlated with manifest exclusion. This supports the second hypothesis, revealing that both physical strength and attractiveness play a role in subtle exclusion. Expanding the scope of prior research, while self-perceived levels of physical abilities and physical condition were negatively related to subtle exclusion victimization [26,29], our findings offer additional insights. Not only are ability and physical condition associated with isolation victimization, but attractiveness and physical strength are also negatively correlated with the levels of subtle exclusion experienced. This broadens the understanding of how various aspects of physical self-concept contribute to feelings of social exclusion in adolescents.

Previous studies on bullying that include social exclusion as one of its forms have shown that the forms of exclusion victimization range from direct social exclusion (manifest exclusion) to more indirect or subtle forms of exclusion, such as malicious rumors through third parties in the peer network (aimed at causing isolation), and that this phenomenon was negatively related to the level of physical self-concept [56,57,58]. In addition, the observation that improving physical self-concept through physical education classes can reduce bullying—including certain forms of social exclusion—[62,63] contributed to the third hypothesis: that the four dimensions of physical self-concept would serve as negative statistical predictors for both manifest and subtle exclusion victimization. This hypothesis was partially supported, as ability, attractiveness, and condition were found to be negative predictors, while physical strength was positively associated with subtle exclusion.

The overall conclusion suggests that the profiles of victims of both forms of social exclusion among peers share poor perceptions of their ability, attractiveness, and physical condition, consistent with earlier findings that adolescents with lower physical fitness or body size are often rejected in competitive or team-based activities [19,20]. However, in the case of subtle exclusion, high self-perception of physical strength appears to predict vulnerability to subtle exclusion. This adds depth to the existing literature, which highlights that poor motor skills and poor physical condition are associated with difficulties in peer relationships and a greater likelihood of being socially isolated [26,29], and that being considered unattractive or having minor physical defects are conditions that greatly increase the likelihood of being rejected and excluded, especially when no effort is made to improve one’s appearance [43]. Therefore, boys and girls with various disabilities or special educational needs related to these domains—abilities, attractiveness, competence, and strength—and who are socially stigmatized by these characteristics, may be more subject to subtle social exclusion among their peers. These findings also emphasize the role of physical education in enhancing self-concept and reducing victimization.

The findings that self-perceived physical strength acts as a positive statistical predictor for subtle exclusion victimization, while not influencing manifest exclusion victimization, offer several inferences. As adolescents mature, displays of physical strength that may cause harm to others are increasingly perceived by peers as inappropriate, disruptive, and undesirable behaviors. Furthermore, when an individual stands out due to qualities such as physical strength, they may become targets of avoidant behaviors from their peers, potentially driven by jealousy or envy [1,57,62]. It is hypothesized that adolescents with a high self-perception of physical strength might be more susceptible to subtle exclusion, as peers may engage in indirect forms of social exclusion—such as ignoring or isolating them—due to feelings of fear or jealousy. This type of exclusion represents a form of indirect aggression, wherein perpetrators perceive less risk in targeting a physically strong individual, as opposed to manifest exclusion, which requires direct confrontation and rejection.

Based on previous studies [54,55,56,57], the fourth hypothesis proposed that in girls, the self-perceived level of physical attractiveness would serve as a negative statistical predictor for both manifest and subtle exclusion victimization. This hypothesis was supported in the case of subtle social exclusion but not for manifest exclusion. The findings indicate that both physical ability and attractiveness negatively predict girls’ likelihood of experiencing subtle social isolation or being ignored. In contrast, the likelihood of experiencing direct social rejection in girls is negatively predicted by their self-perceived physical ability.

Finally, the conclusions of this research also shed light from a gender perspective. While boys and girls are similarly affected by manifest exclusion, girls tend to be more vulnerable to subtle forms of social exclusion. Both girls and boys tend to experience manifest exclusion at comparable rates, with this form of exclusion being overtly expressed among peers through direct rejection aimed at making the victim feel unwanted and undesirable [36,37]. However, girls are particularly susceptible to more subtle and indirect forms of social exclusion [10]. These forms of exclusion often involve socially isolating the victim, spreading malicious rumors within peer groups [35,37], or avoiding interaction with the victim, whether physically, verbally, or visually, in both academic and leisure contexts [27,31].

This study has certain limitations related to its design and methodology. Specifically, the coefficient of determination of the regression is low. Therefore, the results must be interpreted with caution. The cross-sectional approach, while providing valuable insights aligned with the study’s objectives, limits the ability to identify causal relationships. Additionally, although the use of an anonymous self-report questionnaire is one of the most reliable and widely applied methods in victimization research, future studies could benefit from incorporating hetero-reports to achieve data triangulation. Building on the findings and methodology of this research, future investigations could adopt a longitudinal design to explore causal relationships and potential mediation effects. This study also opens new avenues for research, including a deeper examination of social exclusion victimization linked to social stigmas in relation to physical self-concept, physical activity, and physical education. Further studies could also focus on the connection between physical self-concept and outcomes such as self-esteem, wellbeing, or psychological disorders (e.g., anxiety, stress, and depression) in both victims and perpetrators of social exclusion in adolescence. Moreover, designing and testing psychoeducational programs aimed at preventing and reducing both manifest and subtle social exclusion, following the guidelines of this study, would be valuable, especially through longitudinal studies with experimental and control groups.

A comprehensive analysis of this study’s conclusions suggests the need to design and implement psychoeducational programs in schools, starting from preadolescence, that not only encourage physical and sports activities but also foster the development of a balanced and healthy physical self-concept. Such programs could play a significant role in reducing both manifest and subtle forms of social exclusion victimization. Educational interventions should address all domains of physical self-concept—ability, attractiveness, condition, and strength—while placing particular emphasis on enhancing accurate self-perceptions of physical ability and attractiveness, as these are the strongest predictors of social exclusion victimization. Moreover, it is crucial for both general education and physical education to support the balanced development of physical strength, promote self-regulation in behaviors involving physical strength, encourage moral and interpersonal behavior, and teach the appropriate, non-violent use of physical strength in interpersonal and athletic contexts.

Similarly, these psychoeducational programs should incorporate a specific focus on gender. Given that research indicates girls are more affected by subtle social exclusion, with self-perceived physical attractiveness being a significant predictor of this form of exclusion, and prior studies showing that adolescent girls generally have a poorer self-perception of their physical appearance compared to boys [50], and are more vulnerable to concerns related to physical attractiveness, often perceiving themselves as overweight [22], it is recommended that these interventions include learning activities centered on self-perception and body image. These activities should address the aesthetic evaluation of the body, the conception of physical beauty, and the influence of societal and evolving stereotypes. It is essential for all adolescents, particularly girls, to develop socio-critical thinking regarding social stigmas to build positive self-esteem and life satisfaction. This will help them develop independent judgments and resist the pressures of dominant and shifting social stereotypes about body image and beauty. They should be encouraged to cultivate balanced physical development across all domains and to form a realistic and positive self-concept regarding their physical attributes, including ability, attractiveness, condition, and strength. Educational strategies aimed at reducing biases related to appearance, aesthetics, and physical competence [22,23,24,25], while considering a gender perspective, should be applied to all adolescents. Since peer exclusion is an ecological phenomenon, it is crucial to prevent and mitigate it by raising awareness and actively engaging not only potential aggressors and victims but also bystanders.

This study offers a significant contribution by employing a self-report questionnaire specifically designed to assess two distinct subtypes of social exclusion—manifest and subtle—that lack previous precedents in the literature. This tool is unique in its ability to capture both forms of exclusion and is noted for its simplicity and strong psychometric properties, making it an effective instrument for measuring social exclusion victimization among adolescents. Its design aligns with prior categorizations of social exclusion, distinguishing between more overt rejection and more covert isolation [38].

The investigation of social exclusion, both manifest and subtle, during adolescence is a growing field of research. Building upon the established understanding of bullying and cyberbullying, this research has the potential to play a crucial role in addressing victimization during this critical developmental stage. There is an urgent need for further research examining social exclusion in relation to physical condition, physical activity and education, self-perception, and societal stereotypes. Such research is essential for the development of evidence-based prevention strategies and educational interventions that foster positive peer interactions and support the social and individual development of adolescents, thereby reducing associated risks and challenges.

## Figures and Tables

**Table 1:** Linear regression analysis of age, strength PC, attractiveness PC, skill PC, and physical condition PC as predictors of manifest and subtle exclusion.

Variable	Manifest Exclusion		Subtle Exclusion	
	*β*	*t*	*β*	*t*
	R^2^ = 0.051		R^2^ = 0.103	
Age	0.005	0.144	0.045	1.387
PC Strength	0.045	1.157	0.075	2.004 *
PC Attractiveness	−0.093	−2.534 *	−0.130	−3.642 **
PC Skill	−0.195	−5.314 **	−0.210	−5.887 **
PC Physical Condition	−0.027	−0.654 *	−0.119	−2.917 **

*Note*. *p*-Values are indicated according to *p* < 0.01 ** *p* < 0.05 *. *β* = Beta; *t* = *t*-test.

**Table 2:** Spearman correlations between the different physical activity variables and age, manifest exclusion and subtle exclusion.

Variable	Age	Manifest Exclusion	Subtle Exclusion
Manifest Exclusion	0.039	-	-
Subtle Exclusion	−0.076 *	0.783 **	-
PC Strength	−0.025	−0.058	−0.099 **
PC Attractiveness	−0.162 **	−0.184 **	−0.238 **
PC Skill	0.011	−0.233 **	−0.265 **
PC Physical Condition	−0.094 *	−0.150 **	−0.220 **

*Note*. *p*-Values are indicated according to *p* < 0.01 ** *p* < 0.05 *.

**Table 3:** Linear regression analysis by gender, with manifest exclusion as the dependent variable and age, strength PC, attractiveness PC, skill PC, and physical condition PC as independent variable.

Variable	Manifest Exclusion			
	Boys		Girls	
	*β*	*t*	*β*	*t*
	R^2^ = 0.070		R^2^ = 0.032	
Age	−0.047	−1.009	0.050	1.013
PC Strength	0.071	1.385	0.003	0.050
PC Attractiveness	−0.122	−2.391 *	−0.073	−1.390
PC Skill	−0.222	−4.615 **	−0.160	−2.928 *
PC Physical Condition	−0.041	−0.736	−0.013	−0.220

*Note*. *p*-Values are indicated according to *p* < 0.01 ** *p* < 0.05 *. *β* = Beta; *t* = *t*-test.

**Table 4:** Linear regression analysis by gender, with subtle exclusion as the dependent variable and age, strength PC, attractiveness PC, skill PC, and physical condition PC as independent variables.

Variable	Subtle Exclusion			
	Boys		Girls	
	*β*	*t*	*β*	*t*
	R^2^ = 0.099		R^2^ = 0.095	
Age	0.003	0.076	0.084	1.774
PC Strength	0.060	1.188	0.076	1.476
PC Attractiveness	−0.112	−2.245 *	−0.142	−2.806 **
PC Skill	−0.221	−4.663 **	−0.200	−3.787 **
PC Physical condition	−0.137	−2.511 *	−0.085	−1.499

*Note*. *p*-Values are indicated according to *p* < 0.01 ** *p* < 0.05 *. *β* = Beta; *t* = *t*-test.

**Table 5:** Comparison of Manifest and Subtle Exclusion by Gender.

Variable	Girls n = 427	Boys n = 449	*p*	D
	M ± SD	M ± SD		
Manifest Exclusion	1.28 ± 0.49	1.25 ± 0.47	0.119	0.06
Subtle Exclusion	1.50 ± 0.65	1.43 ± 0.57	<0.001	0.11

*Note.* M = Mean; SD = standard deviation; *p* = *p*-value; D = D de Cohen.

**Table 6:** Stepwise regression analysis by gender, with manifest exclusion as the dependent variable and age, strength PC, attractiveness PC, skill PC, and physical condition PC as independent variables.

Manifest Exclusion					
Boys			Girls		
Variables	*β*	*t*	Variables	*β*	*t*
	Step 1 R^2^ = 0.060			Step 1 R^2^ = 0.032	
PC Skill	−0.248 **	−5.423	PC Skill	−0.185 **	−3.876
	Step 2 ΔR^2^ = 0.010				
PC Skill	−0.224 **	−4.796			
PC Attractiveness	−0.114 *	−2.438			

*Note*. *p*-Values are indicated according to *p* < 0.01 ** *p* < 0.05 *. *β* = Beta; *t* = *t*-test.

**Table 7:** Stepwise regression analysis by gender, with subtle exclusion as the dependent variable with age, strength PC, attractiveness PC, skill PC, and physical condition PC as independent variables.

Subtle Exclusion					
Boys			Girls		
Variables	*β*	*t*	Variables	*β*	*t*
	Step 1 R^2^ = 0.073			Step 1 R^2^ = 0.060	
PC Skill	−0.274 **	−6.026	PC Skill	−2.50 **	−5.312
	Step 2 ΔR^2^ = 0.020			Step 2 ΔR^2^ = 0.025	
PC Skill	−0.229 **	−4.869	PC Skill	−0.202 **	−4.194
PC Physical Condition	−0.154 **	−3.266	PC Attractiveness	−0.172 **	−3.572
	Step 3			Step 3	
	ΔR^2^ = 0.007			ΔR^2^ = 0.006	
PC Skill	−0.218 **	−4.616	PC Skill	−0.207 **	−4.296
PC Physical Condition	−0.113 *	−2.230	PC Attractiveness	−0.156 **	−3.208
PC Attractiveness	−0.109 *	−2.199	Age	0.092 *	1.966

*Note*. *p*-Values are indicated according to *p* < 0.01 ** *p* < 0.05 *. *β* = Beta; *t* = *t*-test.

## Data Availability

The data presented in this study are available on request from the corresponding author. The data are not publicly available due to ethical and privacy considerations to protect the confidentiality of participants.

## Appendix A. The **Manifest and Subtle Social Exclusion Scale (ESMASU)**

	**Strongly** **Disagree**	**Disagree**	**Agree**	**Strongly Agree**
1. My classmates make me feel different from them.	SD	D	A	SA
2. I feel undervalued by my classmates.	SD	D	A	SA
3. I feel odd among my classmates.	SD	D	A	SA
4. I feel that my classmates are rejecting me.	SD	D	A	SA
5. My classmates reject me to make me suffer.	SD	D	A	SA
6. I feel like I don’t have a place in the group of classmates.	SD	D	A	SA
7. My classmates exclude me to hurt me.	SD	D	A	SA
8. I feel like my classmates ignore me.	SD	D	A	SA
9. I feel like I’m not welcomed by my classmates.	SD	D	A	SA
10. I feel like my classmates are sidelining or excluding me.	SD	D	A	SA
